# Prospects of using community directed intervention strategy in delivering health services among Fulani Nomads in Enugu State, Nigeria

**DOI:** 10.1186/1475-9276-12-24

**Published:** 2013-04-08

**Authors:** Joseph C Okeibunor, Nkechi G Onyeneho, Obioma C Nwaorgu, Ngozi I’Aronu, Ijeoma Okoye, Felicia U Iremeka, Johannes Sommerfeld

**Affiliations:** 1 Department of Immunization and Vaccine Development (IVD), WHO/AFRO, Brazzaville, Congo; 2 Department of Sociology/Anthropology, University of Nigeria, Nsukka, Enugu State, Nigeria; 3 Department of Parasitology, Nnamdi Azikiwe University, Awka, Anambra State, Nigeria; 4 Humanities Unit, School of General Studies, University of Nigeria, Nsukka, Nigeria; 5 WHO/TDR, Geneva, Switzerland

**Keywords:** Access, Healthcare-delivery, Immunization, Infection, Mortality, Nomad

## Abstract

**Background:**

The Community Directed Interventions (CDI) strategy has proven effective in increasing access to health services in sedentary populations. It remains to be seen if CDI strategy is feasible among nomads given the dearth of demographic and medical data on the nomads. This study thus characterized the nomadic populations in Enugu State, Nigeria and outlined the potentials of implementing CDI among nomads.

**Study design and methods:**

This exploratory study adopted qualitative methods. Forty focus group discussions (FGD) were held with members of 10 nomadic camps in 2 LGAs in Enugu State, as well as their host communities. Thirty in-depth interviews (IDIs) were held with leaders of nomadic camps and sedentary populations. Ten IDIs with traditional healers in the nomadic camps and 14 key informant interviews with health workers and programme officers were also conducted. Documents and maps were reviewed to ascertain the grazing routes of the nomads as well as existing health interventions in the area.

**Results:**

Like sedentary populations, nomads have definable community structures with leaders and followers, which is amenable to implementation of CDI. Nomads move their cattle, in a definite pattern, in search of grass and water. In this movement, the old and vulnerable are left in the camps. The nomads suffer from immunization preventable health problems as their host communities. The priority health problems in relation to CDI include malaria, measles, anemia, and other vaccine preventable infections. However, unlike the sedentary populations, the nomads lack access to health interventions, due to the mutual avoidance between the nomads and the sedentary populations in terms of health services. The later consider the services as mainly theirs. The nomads, however, are desirous of the modern health services and often task themselves to access these modern health services in private for profit health facilities when the need arises.

**Conclusion:**

Given the definable organizational structure of the nomads in Enugu State and their desire for modern health intervention, it is feasible to test the CDI strategy for equitable healthcare delivery among nomads. They are willing and capable to participate actively in their own health programmes with minimal support from professional health workers.

## Introduction

A major challenge for the control of infectious diseases of poverty and the delivery of essential health care is to ensure equity in health care delivery and that health interventions are accessible to all, irrespective of location, socio-economic status and organization, social class, lifestyle or gender. Nomadic populations constitute a significant proportion of the population in many sub-Saharan African countries. In the context of this study, nomadic populations are defined as communities of people that temporarily or permanently move their residence and occupational activities from one location to the other. Nomads include nomadic hunter and gatherers, pastoralists and peri-pathetic communities (i.e., groups of people moving around settled populations and offering a craft or trade). Pastoralists can be further differentiated into a) trans-humans (nomadic groups migrating regularly between two grazing areas along well-defined routes), b) pastoralists migrating along conventional routes but also moving into different areas each year and semi-pastoralists with semi-sedentary residence and mobility patterns [1].

Sixty percent (60%) of the world's estimated 50–100 million nomads and semi-nomads live in Africa. Here, nomadic populations also have the least access to health services when compared with the general population. Access barriers include financial constraints and cultural and political differences between nomadic and settled populations, including health workers. These populations are also disproportionately vulnerable to infectious diseases such as Polio, Malaria, Tuberculosis, Guinea Worm, Leishmaniasis, Onchocercaisis, Intestinal Parasites and Helminths, Brucellosis and Trachoma [1]. The nomad populations in outlying areas face significant challenges regarding access to health services like immunizations. Being located in geographically isolated areas often in small mobile communities with poor roads, where social and economic factors create barriers, they are geographically and socioeconomically hard to reach with effective health interventions especially child immunization against polio and other vaccine preventable diseases.

Little is known of how to best provide essential health care to mobile and remote populations with community involvement. The constant mobility of nomadic populations excludes them, or at the best places them at the edge of health delivery services. The recruitment, training and community support of nomadic community health workers (CHWs) constitute an additional challenge [2,3]. The formal health system seems to be ill-adapted to the nomadic lifestyle. Past efforts to provide health interventions for nomads have proved to be costly and sometimes ineffectual [3,4] due largely to the limited availability of demographic and medical data of the nomads.

It is surmised that improvements in the health of nomads in Africa would depend on better access to health services [5]. All the same opinions differ as to how health services to nomads could best be organized and delivered, giving their mobility and often high schism with their sedentary host populations. In north-west Somalia, nomads wait until all villages in the region are covered by the primary health care (PHC) services, so that nomads can reach services at all times of their movement cycle [6]. However it has been suggested that mass campaign, through mobile units as a temporary measure until permanent services could be set up for the nomads in the Sahil [7].

Ailou (1992) [2] thus argued that it is possible to organize PHC services for nomads. The services should be capable of mobility matching that of the community they serve. They should establish seasonal circuits in accordance with the local patterns of population movements. Integrated fixed and mobile activities should be carried out in each defined operational area. Similarly, Omar (1992) [8] emphasized the need to set PHC programmes for nomadic populations, especially in countries with limited resources and large nomadic communities. Here, it is reasoned that health services for the nomads could be organized with their full involvement in the planning and implementation. As in every PHC programme, the participation of the nomads as community is crucial.

All the same it is noted that involving nomads in their health care may be difficult [4,7,9]. Nomads in Many West African communities, especially Mali were highly suspicious of anything connected to government, feared tax collectors, and avoided gathering in any numbers for the same reason. By contrast, Bentley (1989) [6] suggests that simple outside contacts and regular provision of essential drugs and supplies can be sufficient to motivate communities to help themselves. In Somalia, perception and participation of the community in PHC programmes was better among the northern pastoralists than the southern agriculturalists [10]. In north-east Uganda, one Karimojong pastoralist community successfully managed its own clinic employing staff selected from their midst and using the proceeds to finance small-scale development activities. Similarly, in Southwest Nigeria, the Fulani nomads participated more in health programmes they had access to than their Yoruba host communities [11].

The Community-Directed Intervention (CDI) strategy in which communities themselves direct the planning and implementation of intervention, has been successfully used among settled populations in more than 19 African countries for the annual distribution of ivermectin, a measure to control Onchocerciasis. CDI can also provide an effective platform for the delivery of other health interventions [12] and has proven to be an effective approach for delivering health intervention to resource-poor communities in Africa [13-15]. Among sedentary populations, a recent multi-country study showed CDI to increase access to appropriate treatment with antimalarials and access to ITN and vitamin A supplementation by two-folds. It ensures equity in access to health interventions. However, the CDI approach to health interventions remains to be validated and tested in nomadic populations.

In CDI, the health services and its partners introduce, in a participatory manner, the range of possible interventions, and the means by which the CDI concept can ensure community ownership from the onset. From then on, the community takes charge of the process, usually through a series of community meetings for collective discussion of roles and responsibilities of the community in the CDI process; community decision-making on how, when, where and by whom the intervention will be implemented, how the implementation will be monitored and what, if any, support (financial or otherwise) will be provided to implementers; and collective selection of community implementers. Health workers provide training of community implementers and monitoring but the community directs the intervention process.

However, the prospects of applying the CDI process in the delivery of health interventions to nomadic populations were previously unknown. A study was designed to explore these prospects and determine whether and how the CDI process can be effectively used in the delivery of health interventions in nomadic populations. This paper outlines the potentials of CDI in nomadic populations in Enugu State, Nigeria and characterizes the nomadic populations for the possible implementation of an experimental study on the application of CDI for the delivery of health interventions among nomadic populations.

## Methods

### The study setting

The study was located in Enugu state, in the Southeastern geopolitical zone of Nigeria. Enugu State is located between latitude 6° 30^1^ N and longitude 7° 30^1^ E. It is a mainland state in southeastern Nigeria (http://en.wikipedia.org/wiki/Enugu_State). The state shares borders with Abia and Imo States to the South, Ebonyi State to the East, Benue State to the Northeast, Kogi State to the Northwest and Anambra State to the West.

Lying partly within the semi-tropical rain forest belt of the South, the State spreads towards the North through a land area of approximately 8,727.1 square Kilometres (3,369.6 sq mile). Its physical features change gradually from tropical rain forest to open wood-land and then to Savannah. Apart from a chain of low hills, running through Abakaliki, Ebonyi State in the East to Nsukka in the Northwest, and South-wards through Enugu and Agwu, the rest of the state is made up of low land separated by numerous streams and rivulets, the major ones being the Adada River and the Oji River.

Enugu has good soil and climate, sitting at about 223 metres (730 feet) above sea level, and the soil is well drained. The mean temperature in Enugu State in the hottest month of February is about 97.16°F (36.20°C), while the lowest temperatures occur in the month of November, reaching 68.54°F (20.30°C). The lowest rainfall of about 0.16 cubic centimetres (0.0098 cu in) is normal in February, while the highest is about 35.7 cubic centimetres (2.18 cu in) in July.

**Figure 1:**
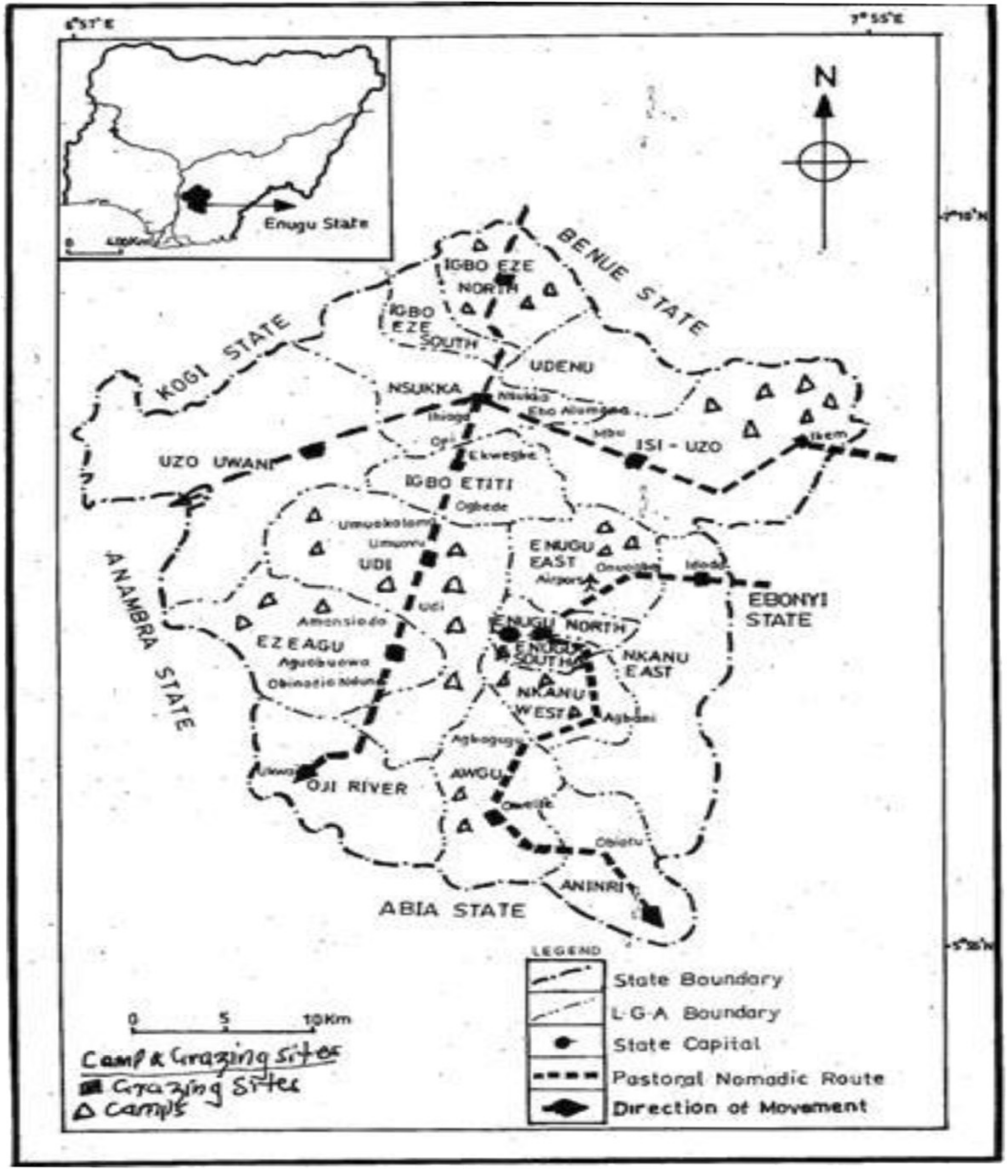
Grazing corridors in Enugu State.

As illustrated in Figure 1, Enugu lies within two grazing corridors for the Fulani pastoralists, coming from the Northwestern geopolitical zone of Nigeria. These are the Enugu-Imo and Enugu-Anambra grazing corridors. The Fulani pastorialists coming from the Northwest geopolitical zone settle in different parts of Enugu State, depending on the availability of grazing land and water for the cattle as well as acceptability of host communities. From here and in their nomadic lifestyle they make optimal use of scarce water and pasture around the streams and rivulets in Enugu State. During the dry season however, they move towards the coastal States of Rivers, Cross Rivers, Bayelsa and Delta States, passing through Anambra, Abia and Imo States.

Though the nomads often avoid exposure to infectious agents by moving away from epidemics such as measles, diseases like trachoma is highly prevalent among them due to flies attracted by cattle; tuberculosis is prevalent due to the presence of cattle, crowded sleeping quarters and lack of health care; treatment compliance is generally poor; guinea worm disease is also common due to unsafe water sources; malaria is usually epidemic, leading to high mortality [1]. Other health problems among these nomads include Leishmaniasis and Onchocerciasis.

Existing health care systems, which promote PHC, with PHC facilities in every community in Enugu State are in the hands of settled populations and rarely extend access to nomads due to cultural, political and economic obstacles. A primary health care system based on nomadic community health workers designed in line with the community directed intervention approach is proposed here. It is suggested that the Fulani nomads are open to modern health care on the condition that it is not an instrument to control them but something they can control by themselves [1]. Similarly, it is reported that Fulani nomads in Western Nigeria complied more to orthodox treatment than their indigenous Yoruba counterparts [11]. The target areas of the study covered two LGAs : Enugu South and Udi.

### Design and scope

This study was exploratory and adopted qualitative methods to learn about the characteristics of nomadic populations in Enugu state; their priority health problems that could be addressed by CDI as well as the existing health interventions among them. The study also documented the delivery processes of existing health intervention and explored the kind of delivery mechanism that will be suitable for CDI approach; explored the mechanisms available for monitoring and evaluation of CDI process as well as mapped the resources available at the community and health system levels for implementation of CDI process. The existing approaches to community mobilization were also documented.

Focus group discussions (FGDs) and in-depth interviews (IDIs) were the main instruments of data collection. Participatory rural appraisal tools were also used in community social resource mapping. FGD sessions were held with community members in the nomadic camps and the host communities. IDIs were held with community and opinion leaders of both the nomadic and host communities; health workers, traditional healers among the nomads. Some key informants in the state capital and Local Government Councils, such as zonal coordinator of the National Council for Nomadic Education, Chairmen of Local Government Councils and the Director of PHC in the Local Government Council and State Ministry of Health were interviewed.

The instruments used in this study were pretested in Nsukka, a town 60–80 km to the study communities, as part of the training of the study team in implementing the study design. This provided opportunity for pretesting the instruments to confirm their (instruments) strength to capture the comprehensiveness of the questions, appropriateness of the sequence of the questions, appropriateness of the duration of the interview, and the potential logistic problems that may arise during the data collection. One pretest IDI each was conducted with a male and a female community leader, for the nomads and residents respectively. One pretest FGD each was also held with male and female groups in the nomadic and sedentary populations. The lessons learnt from the pretest were used to improve the skills of the interviewers and note takers in the IDIs and FGDs.

### Study participants

**Figure 2:**
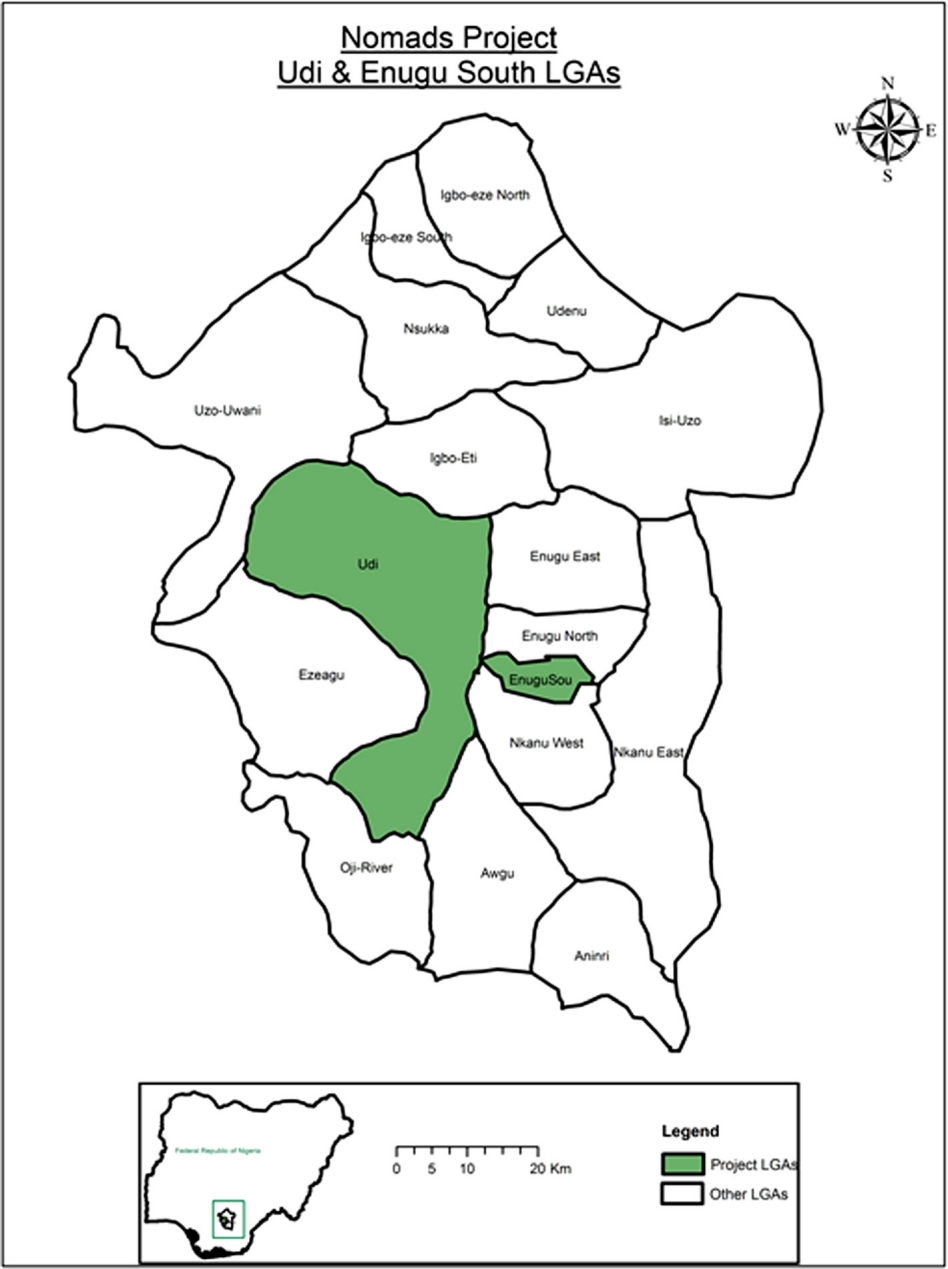
Target LGAs within Enugu State.

The study was conducted in two comparable LGAs, namely Enugu South and Udi Local Government Areas in Enugu State of Nigeria (see Figure 2). In each LGA, 5 communities were randomly sampled to represent nomadic communities of that area. The host sedentary communities were also selected for the study, giving a total of 20 communities in all. See Table 1.

**Table 1:** List of camps and host communities in the study LGAs

**LGA**	**Camps**	**Estimated camp size**^**1**^	**Host community**	**Inclusion**
Enugu South	1. Ardo	Large (est. 600–700 persons)	Akwuke Ukwu	Selected
	2. Nuhu	Large (est. 550–600 persons)	Akwuke Uwani	Selected
	3. Tobe	Medium (est. 300–400 persons)	Ugwuaji	Selected
	4. Gide	Medium (est. 350–450 persons)	Amokwe	Selected
	5. Mangol	Medium (est. 450–500 persons)	Akwuke Ukwu	Not selected
	6. Idris	Medium (est. 300–400 persons)	Aminyi	Selected
	7. Ibrahim	Small (est. 150–200 persons)	Aminyi	Not selected
Udi	1. Haruna	Large (est. 500–600 persons)	Ogwuda Ibude	Selected
	2. Juli	Large (est. 750–800 persons)	Ugwuoji Ngwo	Selected
	3. Lankwoi	Large (est. 600–700 persons)	Ngwo Uno	Selected
	4. Issa	Medium (est. 400–500 persons)	Mgboji	Selected
	5. Bello	Medium (est. 400–500 persons)	Mgboji Uwani	Not selected
	6. Shumo	Medium (est. 450–550 persons)	Mgboji Ngwo	Selected

^1^These figures were collected from the zonal coordinator for National Commission for Nomadic education in the Southeast Geopolitical Zone.

KIIs were held with 14 health professionals. Thirty (30) IDIs were held with camp and community leaders of nomadic and sedentary populations respectively. IDIs were also held with 10 traditional healers in nomadic and sedentary populations. One participatory rural appraisal for social and resource mapping was held in each of the nomadic camps.

A total of forty FGDs were conducted among the nomadic and sedentary populations. The FGDs consisted of 2 sets of female youth and 2 sets of male youth; 2 sets of adult male and 2 sets of adult female as well as 2 sets of elderly males and females. For each set of nomads in FGD, a corresponding set of FGD for sedentary population was also held. Discussions took place under the trees and open clearings or spaces in the camps. In the sedentary communities discussions took place in village squares or palaces of the traditional rulers. Sessions were held throughout the day, from morning until evenings. Participants were purposively selected by the FGD moderator and recorder who walked in different directions from the community or camp centre. Selection and inclusion depended on the group of interest and age of the participants. The FGD participants were full-time residents of the community and camps.

### Data management and analysis

After reviews and corrections, all interview and discussion transcripts were typed with MSWord processing package and converted into American Standard Code for Information Interchange Rich Text Format (RTF) files. These were coded and sorted using the Atlas.ti version 6 programme.

The analysis process began by reviewing the interview and discussion experiences with the field assistants who facilitated and recorded interviews and discussions to obtain their views on the factors that inhibited or animated discussions. A more detailed analysis began with the researchers reading the transcript. During the first reading notes were made of major concepts. A second reading utilized a system of open coding. A re-reading of the texts was done to discern patterns in the ordering and clustering of themes, which provided guide on the systematice development of codes used in Atlas.ti software. This process ensured inter-coder reliability and facilitated triangulation of data from discussions and interviews.

Analysis of the qualitative data placed emphasis on the interpretation, description and recording/writing of what was actually said. The transcripts were first done in the local language and translated into English. In going through the transcriptions, phrases with contextual or special connotations were noted and pulled out as illustrative quotes.

## Results

### Nomadic community and public health context

The nomads comprise of young, adult and elderly men and women numbering between 150 and 800 in each camp. The nomads are predominantly Fulani, and they speak Fulfude. They are also Moslems with little or no formal education. Some of their children however are enrolled in the government supported nomadic education centres located within the camps. The education centres move as the people move and in cases of moving to new areas, new schools are established.

**Figure 3:**
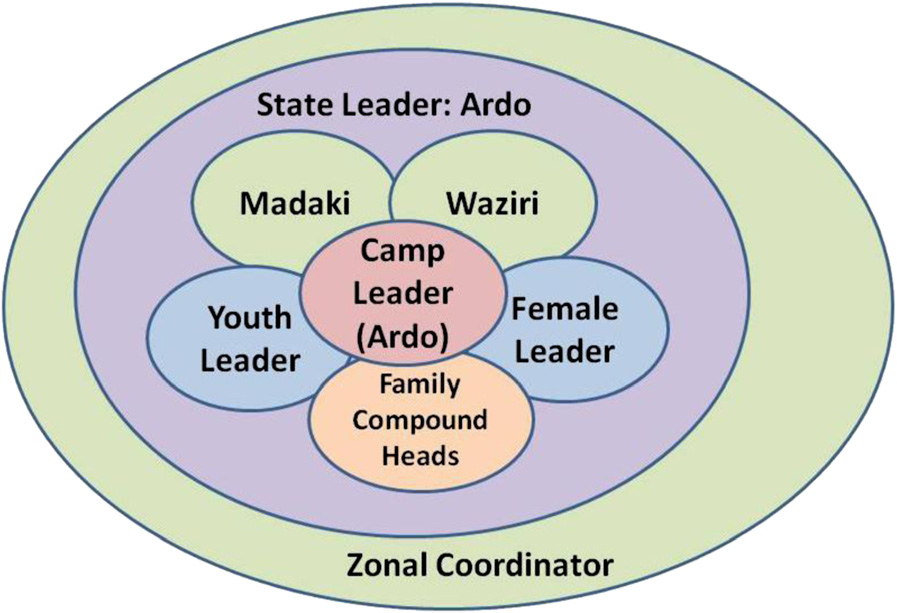
Decision making and leadership structure among the Nomads in Enugu State.

The nomads have well defined leadership structure which extends even beyond Enugu and to the National Association of Nomads with headquarters in Kaduna State, Northwestern Geopolitical zone of Nigeria. Within Enugu state, there is an overall leader of Nomads called the Ardo (see Figure 3). However, each camp has a camp leader who provides leadership with the help of Madaki and Waziri, Chief Adviser and Chief Security Officer. In conjunction with the family heads of each camp, the Ardo and his assistants take decision on the welfare of the camp members. Subsidiary to these are the youth and women leaders, who preside over matters that directly affect the youth or women as the case may be and present same before the committee of elders for further assistance. These could provide structural basis and support for any community directed intervention efforts.

The camps are located in the outskirts of Igbo settled communities who are predominantly Christians. The camps are usually located very close to rivers and rivulets, which never dry up, though may decrease in volume during dry season. The reason for this is not farfetched as they seek areas where water will be readily available for the cattle and also to reduce clashes with sedentary populations, who are predominantly farmers and whose crops would usually be destroyed by grazing cattle. Thus availability of water determines where they camp in Enugu State. However, the availability of grass for grazing dictates the movement away from the water sources. The young men take the cattle in search of grass and could go as far as the Niger Delta region, which is predominantly riverine, in search of grass. As the wet season returns, they also return to the grazing grounds in Enugu State.

**Figure 4:**
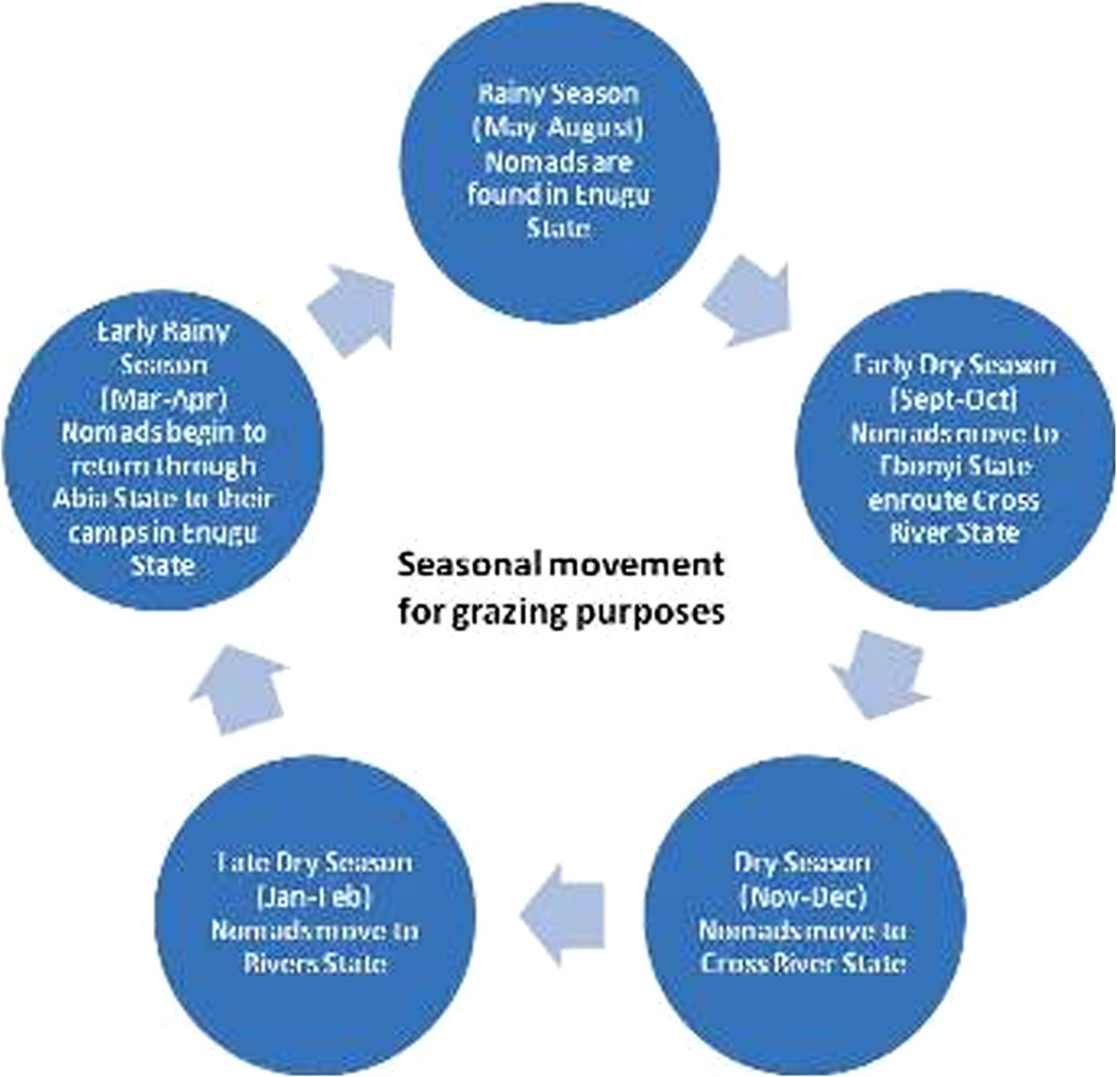
Seasonal grazing movements of the Nomads.

Figure 4 illustrates the nomads' movement in accordance with changing seasons. This is especially in the interest of the animals, which depend on grass for survival. Typically, they graze within Enugu State during the rainy season which is between April and August and begin to move out of the State towards Ebonyi, a neighbouring State and a grazing corridor that leads southwards to Cross River State. As the dry season become stiffer the movement continues further South to Rivers State. The return of the rains again prompts movement from River State through Abia State back to Awgu Local Government Area of Enugu State, which is a major grazing spot. From this point the nomads disperse to their individual camps where they spend the next 4–5 months in Enugu State.

The Igbo sedentary populations have well organized educational and health systems. In all the sedentary communities, there are well established Primary Health Care (PHC) centres. The PHC centres provide the health needs of the community members. Alongside the PHC centres are many profit making health facilities like private clinics, patent medicine vendors (PMV) and even traditional healers.

Unfortunately, the nomads only have access to PMVs and private clinics, which they often refer to as hospitals, when they have need for modern health services. Otherwise, they depend entirely on the traditional healers in their camps for their health needs. One of the health officials interviewed in Enugu South LGA argued that the health commodities and interventions they receive are for their own people and not for others hence they do not cater for the nomadic population. In her words,

…we do not even have enough for our own people talk less of these people we do not know where they come from. The few supplies we have here are used mainly for the indigenes of our local communities and not for strangers. They are not part of us, they just move with their cattle looking for water and grass

Even a top official in the Public Health Directorate of the State Ministry of Health put this more succinctly when he argued that

…we cater for our own people only. These people are nuisance. In my own village we have even driven them away. We are not here to provide for them.... They go around destroying people’s crops with their cattle without caring and when you talk they are ready to fight you....

The nomadic camps are composed of men, women and children with clearly defined roles in terms of who takes care of the cattle, the home and even the health needs of the family. These come out clearly in some quotes during data collection. See a sample quote below:

For females, the roles differ; the roles of the elderly women differ from the roles of the younger females. The elderly women go to the markets and buy some things. They are also responsible for the maintenance of the home. The younger ones would carry the plates to the stream and wash. They would also put on fire for their mothers to prepare the food. The man is the head of the family. He builds the house, takes the cows to the bush and also takes the sick children to the hospital. **[Participant: FGD, Adult Male, Enugu South]**

The Fulani nomads move from different parts of the Northern Nigeria to settle in their different camps in Enugu State. They also move through various LGAs in the State seeking grazing sites and water, down to other states in the South east. Enugu and Ebonyi States provide the points of entry into the vast area of the Southeast and South-south geopolitical zones of Nigeria. Nomads are currently found in over thirty-seven (37) camps in Enugu State. According to the Zonal Coordinator of National Council for Nomadic Education, there are currently 37 camps with nomadic education centres in Enugu State. These camps are currently located in Isi Uzo, Udi, Enugu South and Enugu East, Igbo-Eze North, Ezeagu, Igbo-Etiti Local Government Areas (LGAs) of the States. Previously, they were found in such other LGAs as, Awgu, Uzo Uwani and Udenu as well, but due to conflicts with the sedentary communities they moved to other sites but still return only for grazing purposes. They however notify the zonal coordinator of nomadic activities, with office in Enugu of each move and where they could be located. This is confirmed by Alhaji Ardo, the community leader of one of the nomadic camps and overall leader of nomads in Enugu State. In his words

…anytime we are moving because of conflict with the Igbo community we first of all notify coordinator. He will come to see where we are because of some programmes they have for our children. They have education programmes for our children. This is when we move with our children, women and very old men. Otherwise, the young men keep moving and that is no problem.

The zonal coordinator of nomadic activities in the Southeast of Nigeria confirmed this. According to him,

…it is true that the nomads moved from their old camps to where they are now. But reaching them is not a problem. They are very well organized unfortunately they often have skirmishes with their host communities and have to relocate. But whenever they make any move that closes one camp and to open up another, they inform us. I am always in constant touch with their leaders.

Generally, when the nomads form a camp they tend to remain there for a considerable length of time. The young men move out with the cattle and could go as far as down to other States in the South east and South-south geopolitical zones. The following illustrative quotes elucidate on the mobility patterns of the nomads.

We have stayed here for four years. This year marks the fifth year of our stay. We have not moved because of the school. Our movement disrupts the school activities. That’s why we have not moved. **[Respondent: IDI, Adult male traditional healer, Udi]**

There are those who are semi permanent. They go round in search of pasture while their males take the Cattles to far flung places to graze. But there are those we call the Bororoje. They are a special type of Fulani. You can see them today in Enugu before two or three days they are in Cross River. They are very mobile. But those types that are more or less semi settled a bit and who have reached a compromise with the settled communities, they just go round with their Cattle with its attendant problems. You know that they have huts where they settle their women and young ones…. Well, sometimes three months, sometimes six months and at other times they spend years because they leave their families there and go far for grass when it is dry season here, then when the weather changes, they also return here. **[Respondent: KII, Programme Officer, Ministry of Agriculture, Enugu State]**

The Fulani nomads in Enugu state are well organized with clear structures of relationships and decision making. Those interviewed in the different camps clearly identified their leaderships both for the entire camp and even the women’s groups. They also have defined channels of social relationships with bodies outside the camp and as far as Kaduna where they have the National Association of nomads in Nigeria. The following quote from IDI illustrates the system of leadership among the women in the camps.

We may not have standard mechanism but any problem that comes up in the community as a whole, we as elders gather together and try to see a way of solving it. The ones that happen in the family, the father and the mother handle it, when it becomes difficult, the elders will step in and handle it. **[Respondent: IDI, Female adult, Community leader, Enugu South]**

The male leadership however seems to have a more definite structure. Here one man is the overall camp leader. He is however assisted with a committee of elders and youth in dispensing the leadership needs of the people in the camp. See a sample of quotes which attest this fact.

We have a leader by name Alhaji Ardo and he is assisted by Waziri and Madaki. This is the hierarchy of the leadership structure. In this leadership, Ardo is our overall leader, he attends to every problem, if Ardo is not chanced, he will assign Waziri to attend to the problem, Ardo sends his Madaki to arbitrate in the communities on his behalf. **[Participant: FGD, Male adult, Enugu South]**

Beyond the camp leadership, there is also an overall leader of nomads in the state. This is found in Enugu, the State Capital. In the words of one of the traditional healers

We have one leader in Enugu State. As a leader, I have my cabinet members who oversee the affairs in different camps. However, the members of any camp consult me before they take serious decisions. **[Respondent: IDI, Male adult traditional healer, Enugu South]**

The defined leadership structure notwithstanding, the different segments of the camp communities participate fully in the life of the camp community. The women have ways of getting the men and the leadership of the camp to meet the needs of the women. In most cases, the women meet as a group to discuss the issues, martial out their points which the women leader takes to the male leaders in the camp community. A few argued that the men make decisions and expect the women to comply. For example

If the woman makes decision for the family, the family is gone, there will be no cooperation. It is the man that makes decision for the woman to comply because men are usually careful over making decision that will provoke the anger of a woman. We have to be cautious of making some decisions especially where it concerns the women. There are some aspects the men may decide to involve some of the leaders in the decision making so that acceptable decisions may be reached. **[Respondent: IDI, Male adult traditional healer, Enugu]**

All the same, majority of the people engaged in the process of data collection demonstrated that each segment of the population gets its views considered before decisions that affect the community especially the specific segments are taken. The following quote illustrates this fact.

In decision making, we the women call ourselves if it has anything to do with the women, so that we can take decision about the issue, I personally send my daughter to call my neighbours from their houses.... We the women make decision on things that affect the women **[Participant: FGD, Female Youth, Udi]**

Among the males, everyone is involved both in decision making as well as in protecting the camps.

The Ardo, Waziri and Madaki invite every leader of the various camps and they would gather at a place. Suggestions and opinions would be taken from everybody, at the end the Ardo and the assistants would take a decision.... If it is the decision that affects the Fulani as a community, our leaders meet with the family heads and take a decision, but if it is at home we call elders of each family so that we can sit together and take decision. Members that are drawn from different family units that attended the meeting go back to tell their families. We also use our phones to pass information to each other. **[Respondent: IDI, Male Adult community leader, Enugu South]**

In summary of these points, a traditional healers among the nomads interviewed said,

One person does not take decision that affects others. We as elders come together to take decisions, depending on what the decision is about. Whatever sex or age group is concerned, they are consulted before a final decision is taken. **[Respondent: IDI, Male adult traditional healer, Enugu]**

In terms of the economic life of the nomads and the resources at their disposal, the data revealed that there are so many resources at the disposal of the nomads. Some of the resources include their livestock – cow, goat, sheep and chicken, among others. Those that stay relatively permanent in the camps, while the younger ones take the cattle for grazing, cultivate some crops. There are also products they derive from the livestock such as milk and meat, all of which could be sold when in need of money.

The data also revealed some sex differences in ownership and use of the resources. Generally, it is held that all the resources belong to the husband, who also owns the wife. However, the wife is allowed to use all the resources with the permission of the husband, while she has control over such items as chicken and the milk they get from the cow. Such proceeds from the sale of the milk and chickens are used for providing food for the family. This is supported by the quote from one of the traditional healers from one of the camps in Enugu South. In his words,

The man owns the property and the wife, while the woman owns the husband. 1+1=1. But solely the man owns the property and the woman can easily say it’s her own. If you cooperate with your wife in the area of finance, she will minimize excessive spending of money because she knows your financial strength. I know this because am enlightened and have been a teacher for so many years. I am retired. **[Respondent: IDI, Adult male, Traditional Healer, Enugu South]**

The preeminence of the men over the women in ownership and use of household resources is also attested to by other quotes from community leaders, as well as other male and female members of the community. Below is a sample of such quotes that buttress this phenomenon among the nomads.

The resources of the house are owned by the males but the females have other resources like chicken and they control the proceeds from the sales of such resources. Apart from that one, the men own the cattle and other resources in the family. **[Respondent: IDI, Adult male, Community Leader, Udi]**

In terms of wealth, the men are the owners of the cows. Sometimes women from wealthy homes inherit some cows and they give it to their husbands. If they have any financial needs they can ask their husband to sell their own cows. They also own fowls and cow milk extracted from cow. **[Respondent: IDI, Adult male, Community Leader, Enugu South]**

### Priority health problems and CDI in nomadic populations

In response to the inquiry about the common health problems in the community, the data revealed an inexhaustible list of health problems. According to one of the community leaders interviewed in Enugu South, *“every community has health problems which arise from the degree of feeding. The malnourished ones often get sick. People are generally poor; since they are not feeding well they are prone to develop various degrees of illnesses”*. In line with the views of the community leader, the people listed health problems ranging from communicable diseases like malaria, typhoid, skin diseases, sexually transmitted diseases, and respiratory problems to non communicable diseases like high blood pressure, rheumatism, stroke, among others. There were also problems of anaemia and dehydration among the children.

However, some of the participants argued that the health problems differ with the age of the people in the camps. According to a male youth in Enugu South,

It all depend on the age, the old people suffer from HBP. The youth suffer from tooth ache. We need counselling on how to take care of ourselves.... The youths suffer from AIDS while the adults suffer from High Blood Pressure and other sicknesses.... The major problem in this community is among the children, they suffer from fever, malaria while the old people suffer from high blood pressure and also stroke. Malaria, typhoid, excessive head ache, joint pain and there are some which after rearing our cows disturb us but we don’t know what to call it. **[Participant: FGD, male youth, Enugu South]**

This position was corroborated by other participants in the study. All the same, speaking in general terms, an adult female in Enugu South succinctly stressed that *“malaria is the major health problem....the major problem we have here are malaria, typhoid, arthritis among the elders”*. According to an elderly male in FGD in Enugu South, *“The common health problem is malaria because we have a lot of mosquitoes here. We also suffer from diarrhoea and fever but the most serious is malaria, we lack sufficient portable water and this causes diarrhoea”*.

A community leader says,

The major disease is malaria and for the elderly, hypertension and partial stroke, personally I suffered hypertension six months ago. **[Respondent: Adult male, Community Leader, Udi]**

On the other hand, a traditional healer in one of the camps in Udi said,

The one I normally handle well is pile, syphilis, bacteria, skin infection, and tooth ache, infertility in men, pregnancy, and circumcision. I am a nurse in fact, though traditional medicine. **[Respondent: Adult male, Traditional Healer, Udi]**

An adult community member from Enugu South tried to put time frame to the disease conditions in the communities. According to him, *“the disease that affects us is shortage of blood around August and in the harmattan period. We also suffer shortage of blood alongside malaria*”.

Agreeing with the views of the community members the health workers also prioritized malaria as the most troubling health problem in the communities. In the words of one of the health workers interviewed in Enugu South, *“malaria is of high priority, skin diseases, snake bites and maternal and child health problems”*.

Another health worker interviewed in Udi LGA had this to say,

Mainly during the rainy season, because in the dry season, some of these areas will be dry and there will not be mosquito but in the rainy season, there is plenty mosquito. There was a time we went to one location and malaria had killed two children there that day. I forgot to tell you that because of their location, child delivery is a serious danger. There was this day that we went to another location, a woman died. She died that morning because there was no helping hand. We were so embittered that there was no hospital within walking distance. The distance between their camp and the nearest hospital was not less than thirty kilometers. **[Respondent: Adult male, Health worker, Udi]**

### Delivery processes of existing health interventions

Among the host communities there are health centres and health workers regularly go round to immunize the children against such problems as polio and other preventable childhood diseases. They have also received nets against mosquito bites, among other intervention. Here are a few quotes on the existence of interventions:

The only health intervention we have had is house to house immunization which is for children.... We have been privileged to have immunization people come around but it is for children not for adults or youths. **[Participant: FGD, Female Youth, Enugu South]**

… they immunize our children but I have not seen the mosquito net. I heard over the radio about the distribution of the mosquito net but we have not received any one....There was a time they said they will distribute mosquito net. I don’t know if they finally did it because till today I have not seen any. We have only seen those that do immunization. **[Respondent: KII, Male Adult, Community Leader, Udi]**

The same cannot however be said of the nomads, a majority of whom indicate that they have never received any health intervention. In terms of health interventions among the nomads, there appears to be no clear effort at targeting the nomads for health services. According to a health worker interviewed in Enugu South, when asked of the interventions available for the nomads, she said, “*none that I can tell you is reliable. There was a time they were given immunization but nothing else*. A female community leader in Udi, when asked of any health intervention in her area said, there is *“none at all, not even immunization.... Just like I said earlier, there has not been any intervention of any sort from the government. I still maintain that we have had none”.*

One of the youth engaged in FGD in Udi LGA had this to say,

…what I am requesting the government to do for us, the entire Fulani wherever they are, is that government should get us health facilities. I may not say, come and build us hospital but this like mosquito nets, drugs immunization please they should bring it to us. **[Participant: FGD, Male Youth, Udi]**

A traditional healer in one of the camps in Enugu South stressed that,

Within our settlement we don’t have any health intervention, we don’t have clinic.... We don’t do any intervention here, but I heard that our colleagues in some other camps have benefitted from such programmes....We’ve been here for two years; nobody has brought any intervention here. **[Respondent: IDI, Male Adult, Community leader, Enugu South]**

### Interventions and community involvement: current situation

As seen above health interventions are not available in the nomadic camps. However, in the host communities there are interventions targeting children with immunization. In some communities, nets have been distributed to protect the people against mosquito bites. The nomads thus depend on traditional treatment when they fall sick. According the health workers interviewed the health facilities are far removed from the camps.

It is very far. Not only that they are far, but the terrain is very difficult. You must pull off your shoes, roll up your trousers and walk for more than an hour to get there. You know why they stay in such locations. To reduce clash with the sedentary populations; you know they will need space for Cattle and you know you cannot divorce the nomads from their cattle they cannot do anything without their Cattle. They cannot respond to you except your mission is tied with their Cattle. So that makes them to stay away from the sedentary population and that in itself is a problem for them. **[Respondent: IDI, Adult Female, Health worker, Enugu South]**

The data revealed varying degrees of community involvement in the delivery of the existing interventions in the host communities. In some of the sedentary communities, the people argued that they are not sufficiently involved and that they do not have the needed manpower and other resources to help the delivery of the interventions. The only occasion the nomads in Ardo camp were involved, the nomads participated in mobilizing the women to bring out the children. According to a female youth

Through our leader Alhaji Ardo, they inform our camp elders that they are coming to give such immunization and our elders inform us to stay at home because they are coming. We are not involved in administering the drugs. The only assistance is to bring our children for the officers to give them the drugs or injection. **[Participant: FGD, Female Youth, Udi]**

The nomadic community indicated their willingness to participate, even in contributing resources for the implementation of the programme. They argued that it is for their own benefit and would thus contribute to its success for the sake of their women and children. They noted also that the level of contribution they gave the only instance immunization was brought to them was limited to the demands made of them and that was to bring out children. But if asked to select one of them to go get drugs and distribute they will happily do that. Below are some quote from the nomads:

We would contribute money within ourselves to send the person to go and bring the drugs for us. We would help the person because the person is going to help us collect the drugs. So, we too must help the person. **[Participant: FGD, Male Adult, Udi]**

On the nature of public private partnership in the provision of health care to members of the communities, the results revealed that this is non-existent in the communities studied. Some of the participants in the study indicated that there are traces of NGO efforts, but this is not consistent.

There are none except modern health centre and the health centre is very far from us, we equally lack drugs, government people also neglect us when they come here. There is Global Alliance for Vaccine Immunization. We have NGOs but they are not constant. **[Participant: FGD, Female Elderly, Udi]**

### The potential of a CDI scheme in the nomadic group under study

The study revealed potentials for the implementation of CDI scheme in both the nomadic and sedentary groups. There were some evidence of self help community development projects undertaken by members of the communities. The following quote supports this observation.

We have a lot of community activities that we do go together here....We have community activities that are not related to health. We the people of Uboji built this church in a matter of nine months and we also do a lot of activities together. We have town union and it is through the town meetings that we do a lot of things. **[Participant: FGD, Male Elderly, Enugu South]**

The nomads also demonstrated the same spirit of community development as they cited instances when they organized themselves and invested resources and time for the common good of their camps. The following quotes illustrate the fact of an existence of fora for community based efforts among the nomads.

We don’t have groups but whenever any of us have any problem, the camps would contribute camp by camp and help the person but, we don’t contribute money and keep for community projects. **[Participant: FGD, Male Adult, Udi]**

The health workers interviewed confirmed the claims of the nomads as well as the sedentary populations on the willingness (readiness and capability) of the nomads to support any health intervention that is brought to their camps. For most of the respondents, though the people have no definite rules on how to support interventions brought to them, evidence exist to support the fact that they will do anything they are asked to do in support of health interventions brought to them. At present, the nomads have not been given any specific assignment but have provided minimal support of their own to ensure effective delivery and good coverage for immunization of children. The following quote from the programme officers interviewed illustrate this observation.

Mostly, they see these immunization people moving around so they manage to have their children immunized. There is no special mobilization strategy, but they will co-operate if they are asked to do anything…. There is nothing that is specifically for nomads, so the issue of time of the year does not arise. I think the best they will be to make provision for them specifically. They should be taken care of….There is no clear-cut, well organized, planned health programmes for the nomads or among them. They are on their own, although sometimes, if they stumble on these people doing immunization, they take the opportunity to immunize their own children. **[Respondent: KII; Male Adult, Programme Officer Enugu]**

### Potentials for monitoring and evaluating CDI in nomadic populations

Currently, monitoring and evaluation activities are not common among the nomadic populations. This may be attributed to the fact that there are no systematically implemented health interventions involving the nomadic populations. All the same, the health workers tend to monitor immunization activities in the camps and the nomads had earlier indicated that they are willing to support the health system in any way to ensure the successful delivery of health interventions among their populations. The health workers interviewed also attested to the existence of minimal levels of monitoring involving the nomads. In the words of one of the health workers interviewed

You know what we do here is to monitor. Whenever we are in the field that is what we see. When you get to the camps, they will tell you; someone died yesterday due to malaria, or someone died yesterday while delivering. This is very prevalent. **[Respondent: KII; Male Adult, Health worker, Enugu South]**

### Resources available at the community and health system levels for CDI implementation

With respect to existing and available resources at the community level for the implementation of health interventions in the communities, the majority of the people interviewed noted that they have no such existing human resources like health workers. Among the nomads, it is the sole responsibility of the household to provide for health needs of the individual members. In the words of a female member of one of the camps in an FGD session, *“… we don’t have steady resources, if somebody is sick individually our husband will sell their cow and take the person to the hospital for treatment”*. A male youth from one of the host communities, in an FGD session said that *“there are some nurses from this community that helps them. Some of them help to immunize the.... I don’t see nurses around them* (the nomads)”.

Generally, there is a dearth of resources for the provision of health care in the communities, both for the nomadic and host populations. Consequently, the people have not cultivated traditions of making arrangements of supporting health interventions. According to a male community leader in Enugu South LGA, *“we have no community resources for health care …because there are no facilities there”*. All the same, the nomads as well as the host populations reaffirmed their readiness to support any efforts to promote good health for their people. This is illustrated with the following quote from a nomads:

Just the way you people came, if they come, we try to make them comfortable and offer them whatever we can afford in form of our traditional food. Whatever the Ardo tells us to do, in that aspect, that’s what we do. The order has to come from him. **[Respondent: IDI; Adult male, Community Leader, Enugu South]**

Similarly, both the community leaders of the sedentary populations and health programme officers overseeing the area from the State capital said,

Well, we have what we used in that health centre but the only problem is that they don’t always open. They also call my people for immunization and this has happened for up to 3 times now. **[Respondent: IDI; Adult male, Community Leader, Udi]**

There is a way we are trying to manage the issue of health among this population. The type of school we build now, we want to attach a three bed hospital or clinic to it. This was informed by the number of deaths that we witness. We have been able to do this only in Ikem in Isi uzo LGA. That one is complete but not yet equipped. **[Respondent: IDI; Adult male, Programme Officer, Enugu]**

The people also lacked basic infrastructure and this situation is generally held responsible for the poor health profile of the nomads and even the sedentary communities. This is illustrated by a sample of quotes from discussions.

Because there’s no clean water in the community that’s why they complain about sickness a lot.... There’s no borehole, that’s why people complain about catarrh and all those sickness.... They have not repaired our road that’s why the dust causes catarrh.... We don’t have clean water. **[Participant: FGD; Female Youth, Enugu South]**

The paucity of infrastructure and skilled health workers notwithstanding, the people remain optimistic of the success of any health intervention brought to them. They listed a number of community based organizations that could be mobilized to support such health programmes.

Well, here we don’t have any standing community based intervention programmes rather we keep some drugs in the house. We also contribute money to send the sick person to the hospital if we don’t have any drug to treat the ailment. We keep drugs in our houses since we know the common ailment that affects us.... We don’t have any community based organisation in our own camp but have a general community organisation which involves every Fulani person in Enugu state. **[Respondent: IDI; Adult male, Community Leader, Udi]**

Finally, it was observed that some camps have nomadic education centres. And some of these nomadic education centres have a three bed clinic for management of emergency cases among the nomads. These clinics can be manned by one community health officer trained to attend to the nomads. According to the zonal coordinator of nomadic education in the Southeast geopolitical zone of Nigeria, “*plans are on to build a one bed clinic in all the nomadic education centres to cater for the health needs of the nomads”.* These clinics could also serve as the nucleus for the management of any health interventions among the nomads.

### Existing approaches to community mobilization

There are various mobilization approaches that work with the people, both the nomadic and sedentary populations. These include the use of town criers, religious gatherings, contacting the family heads among others. The use of telephone is also mentioned as an approach for mobilizing members of the communities to action. Mobile phones are readily available in most homes. The following quotes illustrate the existing approaches to community mobilization in the communities studied.

....It is at this meeting that everything about the welfare of this community is discussed. Mobilization on everything is discussed there and decisions are taken. Then among the six camps, each camp will go home and inform the members of their hamlets to participate.... In the mobilization, when there is a programme and it needs others to participate, we pass the information across to the leaders of each of the camps and it is done when we meet at the cattle market because we have a meeting point where almost all the leaders of the settlements meet. Then the leaders go to the camps and inform the head of each family unit about the decisions taken. We also pass information across to our people. **[Respondent: IDI; Adult Male, Traditional Healer, Udi]**

We have town criers and vigilant groups. They make announcement and tell us where the people converge. When we converge, the information would be passed across and those present would brief those that were absent. **[Respondent: IDI; Adult Male, Community Leader, Enugu South]**

## Discussion

The nomads, like other populations in Nigeria, suffer from a myriad of problems ranging from communicable to non communicable diseases. Much like the sedentary populations in Africa, major causes of mortality and morbidity seem to be preventable infectious diseases [1]. This study revealed that malaria, fever, anaemia, skin diseases and snake and insect bites occurred very frequently among the nomads. They blame their condition on their environment. According to one of the nomads in an FGD session, “*as we are in the bush many things bite us especially the women but if it is snake we know the medicine for snake but the other insects we do not know and they give women rashes on their body”.*

As for the children the major problems are malaria fever and anaemia as well as stooling and vomiting. Most mothers complained that each time they take their children to the hospital the health workers complained of the lack of blood in the children’s body. The people consequently request that assistance be given to them to address the persistent problem of malaria and anaemia among their children.

Unfortunately, not much health interventions come to the nomads. They have similar health problems as the sedentary communities but have much less access to health care. This is in line with findings from previous studies on nomads in Africa. For instance, Sheik-Mohamed and Velema, (1999:2) [1] recorded that “in general, nomadic and settled populations in rural Africa are subject to the same kinds of health problems but the frequency of occurrence of specific diseases may greatly differ between nomads and settlers. Nomads appear to be generally healthier than their settled neighbours, but have much less access to health care, safe drinking water and formal education”. In very few cases children in some camps were immunized during the immunization campaign in the sedentary communities. Often the nomads have to go for the health care in private for profit health facilities. Even the health workers do not seem to plan for the nomads. In an interview, a health worker argued that the medical supplies are for their own people (the sedentary population) and not for the strangers (nomads). During the PRA session in one of the camps, a nomad noted that, *“even when we go to the public health centre they will just be looking at us and that is why we do not go there. A hospital is supposed to be for everyone but this is not the case”.* Previous studies have shown that Fulani children had lower immunization rates [16] than the overall population of the LGAs and that Fulani residents were also less likely to be included in LGA guinea worm eradication efforts than Yoruba residents of neighbouring hamlets [17]. However, the important issues in health service utilization among nomads are the preference for private medicine vendors and the avoidance of health facilities, due to mutually avoidance with the host communities [18-21]. The formal health system appears ill-adapted for extending services to constantly mobile communities of nomads [3,18,22] and local authorities often disregard the existence of nomads with respect to health service delivery.

With respect to existing interventions, the study revealed that some interventions like insecticide treated nets and immunization are delivered to the sedentary populations. The sedentary populations are usually involved in the delivery of these interventions at different levels. Some are involved in sensitization and mobilization of eligible persons within their communities. Others participate as local guides, for instance to immunization officers.

However, no such intervention is extended to the nomadic populations in Enugu State. They argued that government should be made to realize that they are also in the country and that they are humans as others thus deserve the same treatment given to the sedentary population.

The study also revealed very high potentials for a CDI scheme in the nomadic groups under study. For instance during the PRA the nomads demonstrated great enthusiasm at the prospects of having one of their own trained to collect and deliver interventions to them in their respective camps. According one of the participants in the PRA, “*why will we not want it? Is it not for our health and that of our children.... In the name of Allah we want it*”. Another queried, “*did you not say we can have one of our own trained to be going to government to collect our own share of drugs?”*

The nomads have a number of resources at their disposal to support the delivery of interventions in their camps. Besides their cows, they also have poultry, goats and other livestock which they could sell to earn money to support their health needs. The women also milk the cows and sell same for the household needs. Sometimes they give proceeds from these as gifts to their host communities. The male groups argued that they could raise money from their cows to support any intervention that is brought to save their lives and those of their children. The women on the other hand indicated that they could sell the milk to raise money in support of the delivery of any health intervention in their camps.

The nomads have young men and women who could be trained to participate in the delivery of the interventions. The elders indicated that they could give the volunteer money for transportation. One of the nomads said, *“… you know our women can easily do this job. We can support them with small something…Some of our wives can speak Igbo now”*. Previous studies in other West African and Sahil societies have also demonstrated that nomads are capable of participating very positively in health programmes if genuinely involved [6,10,11,16,17].

The study also identified potentials for community self monitoring as well as external monitoring of health interventions among the nomads. Within the nomadic populations were elderly men who stay back in the communities at all times. In every camp, there are structures and officials like the Ardo, Waziri and Madaki as well as the committee of family heads, youth and women groupings, all of which could be weaved into a committee to monitor the collection and delivery of health intervention commodities among the populations. Furthermore, the teachers and health workers in the nomadic education centres as well as the three bed clinics in the nomadic education centre provide additional resources for external monitoring and supervision of the implementation process. There are also interpreters from the zonal offices of the National Council for Nomadic Education who could serve as resource persons for the evaluation of the intervention programme. The fact, also that this study is possible attests to the possibility of evaluating CDI in nomadic populations.

Generally the nomadic populations have the full complements of human resources needed for the implementation of CDI in the camps. There are the youth, elderly men and women who could play different roles for the CDI implementation. The Fulani nomads also indicated that they have their cattle, poultry and other animal products which they could sell to support the implementation of the interventions.

Though, there are no health facilities in the camps, the sedentary communities have health facilities with trained workers who could be orientated to provide support for the implementation of CDI among the nomadic populations. Some of the nomads have also picked up the local languages spoken in Enugu State and would not have difficulty going to the health facilities to collect their commodities.

In terms of community mobilization, many approaches are currently being used among the nomads, just like their host communities. Like any Fulani community in Nigeria, the first point of mobilization is to sensitize the camp leader. Because of the leadership structure, the Ardo’s word is law. Once he says come the people come running. Once the Ardo is sufficiently sensitized, he directs other camp leaders. The camp leader in turn directs the Madaki to invite the family heads to a meeting where they are briefed on what has to be done. In cases of emergency, a youth goes from home to home, with the aid of motor cycle inviting people or disseminating whatever information. They also have mobile phones which are usually deployed for mobilization purposes. These are very effective and get immediate response as the nomads are always on their guards against possible aggression from their host communities.

## Conclusion

The study revealed that nomads are generally open to modern health if assured that this will not be an instrument to dominate and control them. The nomads are currently disadvantaged in the provision of health interventions due to physical and psychological distance that exists between them and the sedentary population as well as health officials in the areas. Often the nomads are seen as strangers and not included in the delivery of health interventions. Worse still, they are not included in the censuses of the sedentary population making it difficult for them to be accounted for.

However, the nomads demonstrated enthusiasm and willingness to be involved in the distribution of health interventions as they suffer major health challenges in their daily lives. The nomads also have all the structures that could make the implementation of CDI feasible within their population. The existence of the nomadic education centres, with three bed clinics in each further demonstrates the feasibility of implementing CDI among the nomads, irrespective of their mobility. The mobility patterns of the nomads are well defined and could be worked into the CDI process for maximum results.

## Abbreviations

CDI: Community directed intervention; CHW: Community health worker; FGD: Focus group discussion; IDI: In-depth interview; ITN: Insecticide treated net; LGA: Local government area; PHC: Primary health care; PMV: Patent medicine vendor; RTF: Rich text format

## Competing interests

The authors declare that they have no competing interests.

## Authors’ contributions

JCO and NGO conceived, designed and coordinated the study; OCN and JS reviewed the protocols and improved the intellectual content while NGO, NI, IO and FUI coordinated data collection, analysis and interpreted the data, JCO and NGO contributed to the design of the final manuscript for intellectual quality. The protocol had the ethical approval of the University of Nigeria Teaching Hospital Research Ethics Board and the ethics review committee of the World Health Organization. All authors read and approved the final manuscript.
